# The analysis and quality assessment of translation strategies in subtitling culturally specific references: *Feathers*

**DOI:** 10.1016/j.heliyon.2023.e19095

**Published:** 2023-08-11

**Authors:** Ahmed M. Alaa, Islam Al Sawi

**Affiliations:** a The College of Language and Communication (CLC), The Arab Academy for Science, Technology and Maritime Transport (AASTMT), Egypt; b The Faculty of Languages, Modern Sciences and Arts University (MSA), Egypt

**Keywords:** Translation, Subtitling, Culturally specific/bound references, Arabic to English

## Abstract

Subtitling poses significant challenges, particularly when dealing with culturally specific/bound source texts (STs). This research paper aims to identify and assess the strategies employed by professional translators in rendering culturally specific references from Egyptian Arabic into English in the award-winning Egyptian movie, *Feathers*. In this study, Pedersen's (2011) typology of translation strategies was utilized to identify the strategies employed, while Pedersen's (2017) functional equivalence, acceptability, and readability (FAR) model was employed to assess the quality of the subtitles. The findings revealed the utilization of Pedersen's suggested strategies, excluding the use of official equivalents, with a prevalent adoption of target-text-oriented (TT-oriented) strategies, such as substitution, generalization, and omission. Additionally, the results indicated that formality represents an additional translation strategy that coexists with other strategies, rather than being a media-specific constraint. Moreover, the quality assessment demonstrated that the majority of the subtitles exhibited high quality, with only a few minor errors observed, primarily related to functionality.

## 1 Introduction

Subtitling has emerged as an indispensable tool in our interconnected world. As our societies become more globalized, individuals are increasingly exposed to diverse communities and cultures. To bridge the gap between these cultures portrayed through media, subtitling plays a crucial role by providing translated text on screen for audiences to read and comprehend. Subtitling is defined as

a translation practice that consists of rendering in writing, usually at the bottom of the screen, the translation into a target language of the original dialogue exchanges uttered by different speakers, as well as all other verbal information that appears written on screen (letters, banners, inserts) or is transmitted aurally in the soundtrack (song lyrics, voices off) [1, p. 274].

Subtitling can be either intralingual, where subtitles are written in the same language as the spoken script, or interlingual, where subtitles are a translation of the spoken script into another language. Subtitling also differs from other types of audiovisual translation in that it is written and keeps the source dialogue, unlike dubbing and voiceover, for example, which are oral and remove the source dialogue. Moreover, subtitling is governed by two main factors: space and time. Each subtitle should fit the limited space available on the screen and be in synchrony with the speech [1,2].

Malenova states that four main factors may restrict the translator's work on subtitling. Normative restrictions focus on achieving equivalence between the source text (ST) and target text (TT). Social restrictions necessitate that cultural, moral, ethical, and religious values be taken into account. Personal restrictions are influenced by the translator's worldview and might result in unusual translation decisions or mistakes. Last, physiological restrictions pertain to how subtitles are presented, which entails following specific principles for subtitling, such as the use of simple grammatical units, self-contained subtitles, and a restricted length of characters [3].

Moreover, Malenova notes that in order to provide user-oriented translations that maintain the message and meaning of the ST, the translator must be cognizant of intertextuality and culturally specific references to be able to render them correctly in the TT [3]. Diaz-Cintas and Remael define these culturally specific references as “extralinguistic references to items that are tied up with a country's culture, history, or geography, and tend therefore to pose serious translation challenges” [2, p. 200]. Therefore, translating these references can prove challenging because of the absence of an equivalent in the culture of the target audience and the limited space available for each subtitle. Zojer adds that due to the many restrictions imposed on subtitling, there may not be a standard agreed-upon translation, where the subtitle may show radical changes from the original speech to make it comprehensible to the target audience. This requires the translator to employ a set of strategies to render successfully the source culture into the target one [4].

Thus, there is a need to identify, analyze, and assess the strategies translators follow in subtitling culturally specific references. This will help translators sharpen their skills when dealing with such challenging instances in translating texts, in general, and in subtitling, in particular. There were some attempts to develop a framework of translation strategies to guide translators in subtitling culturally specific references. This study selects Pedersen's (2011) framework due to the following points of strength: (a) it is a recent framework that builds on the work of previous frameworks and scholars; (b) it is supported by empirical research and it witnessed some updates, reaching the latest 2011 version that is more accurate and comprehensive; (c) it is simple and easy to use, with well-constructed, clear-cut categories; (d) it fits the current study's objective of investigating the semantic transfer of cultural references from one language to another. This framework is discussed in detail in Section 3.

## 2 Empirical studies on subtitling cultural references in movies

Cultural references are of a problematic and challenging nature across different languages/cultures. Several studies have employed various frameworks to identify the diverse strategies used for subtitling cultural references in movies of varied genres. The following review of the literature focuses on selected papers to elucidate this.

To start with, translation studies from English to Lithuanian showed that translators may use various typologies to identify translation strategies used to subtitle culturally specific references in different movie genres. Judickaitė studied the strategies used to translate the cartoon *Ratatouille* from English to Lithuanian using Tory's (1995) coupled pairs. The results showed that translators were inclined to domesticate cultural references into the TT by means of globalization to help make the references comprehensible to the audience [5]. Moreover, Horbačauskienė et al. used the typology proposed by Pedersen (2011) for culturally specific references' translation from English into Lithuanian in the Australian TV reality show *My Kitchen Rules*. They concluded that translators opted for source-oriented transference via retention because it is an easier way to deal with cultural references, especially with amateur translators, but it may result in an inadequate quality of translation [6]. Additionally, Iliavičius used Pedersen's (2011) taxonomy to investigate the translation strategies of cultural references used in five English science-fiction movies: *Edge of Tomorrow, Arrival, The Martian, Interstellar,* and *Inception*. Results indicated that source-oriented translation strategies were the most often used, with retention and official equivalent being the most frequently used translation strategies when rendering culturally specific references. However, unlike Horbačauskienė et al., Iliavičius did not generally see that as a weakness [7].

Similarly, articles focusing on subtitling cultural references from English to Chinese/Chinese to English showed that subtitling cultural references is a challenging task that requires special cultural knowledge from the part of translators and hence is worth researching. Zhao employed the taxonomy proposed by Aixelá (1996) to translate cultural references to investigate the strategies used in translating the American sitcom *Friends* to Chinese. He concluded that repetition is not an effective strategy despite being loyal to the ST. He recommended the use of substitution, creation, and deletion as alternatives to repetition to help produce a subtitle familiar to the Chinese audience [8]. Moreover, Lau et al. adopted Newmark's (1988) cultural references' classification and Aixela's (1996) strategies for translating cultural references to identify the strategies used in Chinese to English translation in the martial arts movie *Crouching Tiger, Hidden Dragon*. The findings revealed that translators did not have to translate many cultural references because the movie's nonverbal features, such as gestures, could help explain the meaning vividly. They recommended that if visual actions are unable to express cultural references, translators have to be aware of the Chinese and English norms to be able to use effective strategies to simplify these references [9]. Additionally, Chai et al. aimed to identify the strategies and challenges faced while subtitling a Chinese cultural documentary on the art of paper-cutting, entitled *The Life of Paper-cutting,* using Tomaszkiewicz's (2010) subtitling techniques. The results showed that the most appropriate translation strategies were omission, direct transfer, equivalence, and adaptation, with retention as the most frequently used strategy. The researchers concluded that to avoid the main challenges identified, whether technical, cultural, or linguistic, translators must be fully aware of the Chinese and English cultures [10]. These papers indicated the importance of effective subtitling of cultural references to bridge the gap between the English and Chinese cultures, but they did not have consistent results when it came to the framework used or strategies employed to translate cultural references.

The same stress on the problematic nature and diverse strategies of subtitling cultural references was evident in studies focusing on subtitling from English to Arabic. Debbas and Haider investigated the strategies used to subtitle religious terms, jokes, and humor in the English cartoon series *Family Guy* for the Arab audience. The analysis revealed that translators tended to use omission with religious and taboo terms. However, with humor and jokes' subtitling, they used three strategies: retention, retention with guidance, and retention with detailed explanation. They concluded that the target audience can influence determining the appropriate translation strategies according to their ideology and culture [11]. In addition, Abdelaal studied the American movie *The American Pie* to investigate and assess the strategies used to render cultural references from English to Arabic using Pedersen's (2011) typology and Pedersen's (2017) functional equivalence, acceptability, and readability (FAR) model. Abdelaal confirmed the use of Pedersen's strategies and suggested the addition of euphemism and formality as extra two strategies to render cultural references. He added that most of the subtitles were of good quality, a claim that can be argued against because of his dependence on fansubs without acknowledging them as a unique genre [12].

Despite their scarcity, few papers attempted to examine the subtitling strategies used to translate cultural references from Arabic to English. Haider et al. argued that translating from a conservative culture, Arabic, to a liberal culture, English, would require the use of specific subtitling strategies to render cultural references successfully. Using Ljung's (2011) typology, they explored the translators' strategies used in subtitling taboo words in the Jordanian Arabic vernacular series *Jinn* into English. The results revealed that translators opted for three strategies: translating a taboo term by another taboo term in the TT; deleting the ST taboo; and, surprisingly since English is considered by the researchers as a liberal language, using a euphemistic TT term. The researchers concluded that further studies on translating Arabic to English cultural references are needed [13]. Another study focusing on translating cultural references from Jordanian Arabic to English is written by Mehawesh and Neimneh. Using the strategies proposed by Gottlieb (1992), they studied the Jordanian Bedouin movie *Theeb*. The results showed that translators used condensation, decimation, transfer, imitation, and deletion but ignored other strategies when rendering cultural references from Arabic to English. The researchers also indicated that because translators opted for functionality, they ignored much of the semantic content present in the ST and hence failed to transfer the Islamic culture to the TT audience. The researchers concluded that there is a need for studies on audiovisual translation, particularly translating dialect, and urged for further research in this regard [14].

It is probably worth noting that Egyptian Arabic exhibits distinct lexical, morphological, and phonological features when compared to Standard Arabic or other varieties found in the Arab World. In terms of vocabulary, Egyptian Arabic incorporates loanwords from various source languages. For instance, words such as “pepsi” and “cancel” have been assimilated into the lexicon. Morphologically, Egyptians tend to integrate these loanwords into Arabic, resulting in derivational changes like “nicancel” (we cancel). Furthermore, it is worth noting that Egyptian Arabic commonly replaces the Qaf sound /ˈq/ with a glottal stop /ʔ/ [15]. These linguistic characteristics are a few examples that highlight the uniqueness of Egyptian Arabic and emphasize the importance of having translators who possess proficiency in both Egyptian Arabic and English to accurately convey meaning, tone, and cultural context.

The literature reviewed indicates the following. First, subtitling cultural references is challenging and therefore is worth researching. Second, the role of a translator is noble and s/he should always attempt to bridge gaps, if any, between cultures and languages, by being culturally sensitive. Third, it seems that the framework used, time and space constraints of subtitling, the distance/similarities between the ST and TT cultures, the movie genre, and other features may create singular conditions for subtitling cultural references in movies, hence the diverse results reported after studying movies' cultural references’ subtitling. Finally, studies on subtitling Arabic dialects into English are scarce and further research is needed. Because of these reasons, this study aims to

1. Identify the strategies used in subtitling cultural references from Egyptian Arabic to English;
2. Assess the translation quality of the cultural references' subtitles.

Hence, the study attempts to address the following research questions:

1. What are the strategies used in subtitling cultural references from Egyptian Arabic into English in the award-winning Egyptian movie *Feathers* following Pedersen's (2011) typology?
2. To what extent were the cultural references' subtitles functionally equivalent, acceptable, and readable according to Pedersen's (2017) FAR model?

## 3 Theoretical framework

This study adopts a qualitative approach to identify and assess the quality of the translation strategies used to translate culturally oriented references in the movie *Feathers*. This study adheres to the qualitative interpretive research model, which is relevant to descriptive translation studies.

The study used Pedersen's (2011) typology of translating culturally specific references, and the quality of the translation was assessed using Pedersen's (2017) FAR model.

Pedersen's (2011) model for translating culturally specific references is deemed the most comprehensive and hence frequently used in subtitling research studies (see Refs. [6,12,16]). He identified seven baseline categories: retention, specification, direct translation, generalization, substitution, omission, and official equivalent, and defined them as follows:

*Retention*. Here the ST ECR is retained in the subtitle unchanged, or slightly adapted to meet TL requirements.

*Specification.* More information is added, making the subtitled ECR more specific than the ST ECR. This is done by completing or fleshing out a name or an acronym (Completion) or by adding more semantic content.

Direct Translation. The only thing that gets changed using this strategy is the language; no semantic alteration is made.

*Generalization*. This strategy makes the TT rendering less specific than the ST ECR. It can be done either by using a Superordinate Term or a Paraphrase.

*Substitution*. The ST ECR is replaced by another ECR, either from the SC or the TC. Alternatively, the ECR could be replaced by something completely different.

*Omission.* The ST ECR is not reproduced in any way in the TT.

*Official Equivalent*. Either through common usage or by some administrative decision, a SC ECR may have a ready-made Official TL Equivalent [17, p. 76].

Additionally, Pedersen proposed a provisional model to assess the quality of interlingual subtitles, though elusive and too complex to measure. Pedersen's FAR helps pinpoint in writing the knowledge that professionals use to assess subtitles. It is a tripartite model assessing functionality, acceptability, and readability using error analysis and penalty points. Functional equivalence refers to “how well the message or meaning is rendered in the subtitled translation” [18, p. 217]. It assesses how far the subtitle could convey the utterance and its meaning. Errors under this category could involve semantic errors, where the meaning is not maintained, or stylistic errors, which are related to register and style. Semantic errors could get a penalty of 0.5 for minor errors, mostly lexical, not affecting the movie plot, 1 for standard errors, defined as an error that “has bearing on the actual meaning and does not seriously hamper the viewers' progress beyond that single subtitle” [18, p. 219], and 2 for serious errors hindering understanding and causing viewers' frustration. Stylistic errors are less serious than their semantic counterparts, taking the scores of 0.25 for minor errors, 0.5 for standard errors, and 1 for serious ones.

Secondly, acceptability is about “how well the target text conforms to target language norms” [18, p. 220]. Errors under this class can involve grammar, spelling, or idiomaticity errors, where the native-like selection of the subtitle is not achieved, resulting in unnaturalness. Errors under grammar are either minor, if only bothering purists, serious, if they hamper understanding, or standard if in between. Spelling errors are measured based on their seriousness: minor for common errors, standard for spelling errors changing the meaning of the word, and serious for the ones deterring understanding.

Finally, readability involves errors with segmentation and spotting (i.e., flow and synchronization), punctuation, and reading speed and line length. Errors in readability are related to subtitles that are not synchronized with speech, confusing use of punctuation and graphics, and unsuitable subtitling length and speed that make the text hard to follow.

**Fig. 1:**
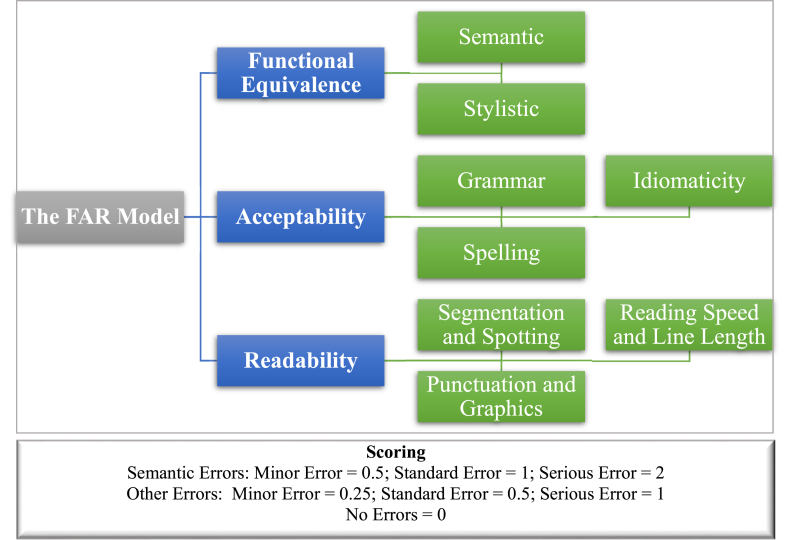
The FAR model.

The FAR model was selected because of its focus on subtitling unlike other general translation assessment models that are “difficult to adapt to the special conditions of the medium [subtitling]” [18, p. 212]. It is also suitable because it looks at the finalized product rather than the process, despite the process being conditional on product quality. The present application of the FAR on the subtitling of Egyptian Arabic culturally specific references into English to assess their quality represents one of the very initial attempts. Fig. 1 illustrates the FAR model.

## 4 Data

Data was collected in alignment with the study's aims and objectives. In order to show the subtitling strategies professional translators adopt to render culturally specific references, the researchers had a list of suggested Egyptian movies that have clear representations of cultural expressions and practices. They then selected the movie *Feathers* for the following reasons: (a) it is an award-winning, globally recognized movie, which proves its quality and authentic content; (b) it has several instances of cultural references, which enriches the analysis and the study outcomes; (c) it is subtitled by a professional agency, which adds to the reliability of the translation provided. After that, each researcher watched the movie more than once and accurately pinpointed each cultural reference depending on the knowledge of the authors, being natives of Egyptian Arabic and proficient speakers of English, and took note of the translation provided to be analyzed for the translation strategy used and the quality of the translation.

Moreover, to enhance the reliability of the data analysis, an inter-rater check was employed. Each of the authors independently analyzed the data according to the subtitling strategy used and the quality of the translation, using Pedersen's (2011, 2017) models. Then, a professional translator/linguist was hired to do the same task. No major differences were found and a decision was then made by the authors concerning each subtitle based on the agreement of at least two of the three raters.

### 4.1 The movie

The movie *Feathers* tells the story of a woman who is forced into a period of self-discovery after her authoritarian husband is accidently turned into a chicken by a magician during their younger child's birthday party. The drama is written by Ahmed Amer and Omar El Zohairy, directed by Omar El Zohairy, and features Demyana Nassar, Samy Bassiouny, and Fady Mina Fawzy. *Feathers* was the first Egyptian movie to win the 2021 Critics' Week top prize at Cannes. The movie was celebrated by critics for “revealing a lot about the Egyptian society” [19]; being “a beautifully framed, sharply observed, quiet depiction of social inequity, squalor and the subjugation of women in an Egyptian factory town” [20]; and shedding light on “the pitch-black (…) working class in an industrial Egyptian nightmare” [21]. Hence, the movie was selected for its international recognition and use of many culturally specific references pertinent to Egyptian society's food, domestic life, and slang/colloquial style.

### 4.2 The subtitles

One point of weakness found in recently published translation papers is the use of fansubs without distinguishing them from subtitles done by paid professionals (e.g. Ref. [12]). Wilcock [22, p. 3] confirmed that fansubbing is a form of subtitling written by fans often with little or no training. He added that their subtitles “can differ substantially from those created in a professional environment. For this reason, fansubs could be seen as a new genre of subtitling, employing strategies that would be unacceptable in a professional subtitled product aimed at general consumption.” The same distinction between subtitles and fansubbing was shared by more researchers (see Ref. [23]). To overcome this issue, and to collect English subtitles suitable for classification and assessment, the data was based on the professional subtitles made by Haghefilm Digitaal, a professional, “specialist postproduction house and motion picture film laboratory offering end-to-end services, film restoration and consultation” since 1899 [24]. The data consisted of 68 culturally specific subtitles.

## 5 Results and discussion

This section highlights the translation strategies found in the analyzed subtitles according to Pedersen's (2011) model. It also explains and discusses these strategies and assesses their quality according to Pedersen's (2017) FAR model.

### 5.1 Retention

Opposite to what Pedersen considered to be “the most common strategy for rendering ECRs” [17, p. 78], retention was only used twice in the subtitles under analysis. This could be because the translator was loyal to the TT; that is, by avoiding retention, the translator could use other strategies that may help domesticate the ST cultural reference, which can explain the pervasive use of substitution for cultural and situational purposes in the subtitles. It could also be because the movie did not name its characters and hence the retention of proper nouns was not needed.

The two occurrences of retention (Table 1) involved slight adjustments to meet the TL conventions with one noun marked with inverted commas and another unmarked. Both subtitles show the translator's loyalty to the ST, which is rare in the data analyzed, and fidelity to the TT. This is because “يلا حالا بالا بالا حيوا أبو الفصاد” (Let's now cheer for Abu al-fasad) and “هات كوكاكولا تانية” (Bring another Coca-Cola) are very unlikely to happen in this form in either situation, a birthday or ordering a soft drink. Therefore, by allowing the nouns “Abu al-fasad” and, to a lesser degree, “Coca-Cola”, the translator allows foreign terms to enter the TT without providing guidance to the target audience.

**Table 1:** Retention.

Time	Arabic	English Subtitle	Back Translation
14:07	سنة حلوة يا جميل، سنة حلوة يا جميل، يلا حالا بالا بالا حيوا أبو الفصاد	Happy birthday to you. Let's celebrate “Abu al-fasad's” birthday now	Have a good year you beautiful, have a good year you beautiful, let's now cheer for Abu al-fasad.
52:06	هات كوكاكولا تانية هنا يابني	Give me another Coca-Cola	Bring another Coca-Cola here, my son.

Table 2 summarizes the quality assessment of the subtitles where retention is used. As seen in the table, the translation is mostly of good quality, with only two minor errors. The first minor error is in semantic equivalence, where the retention of “Abu al-fasad” does not explain the allusion behind using this particular proper noun in this particular context. The other minor error is in the same subtitle, but in relation to idiomaticity, where the use of “Abu al-fasad” in this context may sound odd. Despite that, the general meaning of the subtitle remains clear, reflecting a birthday celebration.

**Table 2:** Quality assessment of the subtitles translated by retention.

Arabic	English Subtitle	Functional Equivalence	Acceptability	Readability
سنة حلوة يا جميل، سنة حلوة يا جميل، يلا حالا بالا بالا حيوا أبو الفصاد	Happy birthday to you. Let's celebrate “Abu al-fasad's” birthday now	0.5 (semantic)	0.25 (idiomaticity)	0
هات كوكاكولا تانية هنا يابني	Give me another Coca-Cola	0	0	0

### 5.2 Specification

By adding information that is not present in the ST to explain a cultural reference, the translator interferes to make an ST cultural reference accessible to the target audience [25]. Table 3 shows how the translator added semantic/linguistic content to the ST references to make them more specific. In “انهارده وبكرة بتنجان” (Today and tomorrow eggplant), the translator adds information to refer to a famous dish in Egypt (We're having fried eggplant today and tomorrow). Another example is when the translator added information to define the meaning of “يلا حالا بالا بالا حيوا أبو الفصاد” (Let's now cheer for Abu al-fasad) to become “Let's celebrate “Abu al-fasad's” birthday now”, specifying the celebration to be of a birthday. Moreover, by adding the official form of request in English (We'd like …) and explaining “حاجة ساقعة” (something cold) as “cold drinks”, the reader could clarify that the actors were trying to order food and explain the kind of drinks they wanted. The same strategy was used to explain the word “بعيد” (far) as “somewhere else” and “الجماعة” (group) as “my friends” to remove any ambiguity among the TT audience because of the ST culturally specific references.

**Table 3:** Specification.

Time	Arabic	English Subtitle	Back Translation
4:16	انهارده وبكرة بتنجان	We're having fried eggplant today and tomorrow	Today and tomorrow eggplant.
11:21	العب بعيد يلا!	Go play somewhere else, boy	Play far away, boy.
14:07	سنة حلوة يا جميل، سنة حلوة يا جميل، يلا حالا بالا بالا حيوا أبو الفصاد	Happy birthday to you. Let's celebrate “Abu al-fasad's” birthday now	Have a good year you beautiful, have a good year you beautiful, let's now cheer for Abu al-fasad.
44:42	4 برجر وحاجة ساقعة. حاضر يا ريس	We'd like four hamburgers and cold drinks. Yes, sir	4 burgers and something cold. Yes, Boss.
52:40	هقعد شوية مع الجماعة بتوعي	I'll be there with my friends	I'll sit a bit with my group.

Table 4 summarizes the quality assessment of the subtitles where specification is used. Generally, the subtitles are well presented and reflect the desired meaning clearly. The only exception is in the translation of “يلا حالا بالا بالا حيوا أبو الفصاد” (Let's celebrate “Abu al-fasad's” birthday now), which, as explained previously, shows minor equivalence and acceptability errors. In addition, there is a minor semantic equivalence error in translating “برجر وحاجة ساقعة 4”, where “hamburgers” is used instead of the more accurate “burgers”. This is still a difference in meaning that can go unnoticed by the reader.

**Table 4:** Quality assessment of the subtitles translated by specification.

Arabic	English Subtitle	Functional Equivalence	Acceptability	Readability
انهارده وبكرة بتنجان	We're having fried eggplant today and tomorrow	0	0	0
العب بعيد يلا!	Go play somewhere else, boy	0	0	0
سنة حلوة يا جميل، سنة حلوة يا جميل، يلا حالا بالا بالا حيوا أبو الفصاد	Happy birthday to you. Let's celebrate “Abu al-fasad's” birthday now	0.5 (semantic)	0.25 (idiomaticity)	0
4 برجر وحاجة ساقعة. حاضر يا ريس	We'd like four hamburgers and cold drinks. Yes, sir	0.5 (semantic)	0	0
هقعد شوية مع الجماعة بتوعي	I'll be there with my friends	0	0	0

### 5.3 Direct translation

Unlike other translation strategies, direct translation does not involve any changes to the semantic load of the ST when rendered to the TT. The translator neither tries to change nor attempts to “transfer connotations or guide the TT audience in any way” [25, p. 5]. The problem with adopting this strategy rises when the translated term is culturally bound. To illustrate, such cultural references may allude to meanings that are not present if literally translated, especially when translated to a culture very different from the ST's [26].

Direct translation was employed exclusively to translate allusions in the movie. Allusions are “generally regarded as brief but purposeful references, within a literary text, to a person, place, event, or to another work of literature” [27, Para. 2]. As seen in Table 5, by adopting this strategy, the translator seems to, either intentionally or unintentionally, sacrifice rendering the references and meanings these allusions make. The allusion in “يا ترى انت فين يا مرزوق؟” (Where are you, Marzouq?) refers to the story of Marzouq Al-Ataki, the hero of the radio operetta (Oath and Arzak) in the 1960s in Egypt, rather than the hero in the movie who has no name, whose wife used to ask, “Where are you, Marzouq?” until he came back very rich. The allusion here hints at the movie heroine's future quest to search for her missing husband who adversely comes back sick and filthy. The direct translation seems to drop the cultural reference and its intertextual functions.

**Table 5:** Direct translation.

Time	Arabic	English Subtitle	Back Translation
17:31	يا ريتني كنت معاهم	I wish I were with them	I wish I were with them.
17:40	كنت عملت عمايل	I'd have done many things	I'd have done many things.
17:54	يا ترى انت فين يا مرزوق؟	Where are you, Marzouq?	Where are you, Marzouq?

Similarly, the expressions “يا ريتني كنت معاهم” (I wish I were with them) and “كنت عملت عمايل” (I'd have done many things) are culturally bound references where the former, originally a quote from a popular Egyptian military movie, *Al-Tareek Ela Eilat* (Road to Eilat), is used sarcastically to refer to situations that begin badly but end well (see Twitter # “يا ريتني كنت معاهم” to make fun of the hijacked plane incident in 2016, e.g.), and the latter is used in many Egyptian plays and movies for its humorous effect. Direct translation may have failed to render the intertextual reference and connotative meanings into the TT; however, it could be justified by the complex nature of rendering allusions in subtitles between two different cultures/languages and by the utterances being said by a minor character who did not appear on screen but could only be heard.

Table 6 summarizes the quality assessment of the subtitles where direct translation is used. As shown in the table, a common error in the three direct translation instances in the data is that the translator misses transferring the origin of the allusion expressions in the ST to the TT. However, due to the limited space and time available for each subtitle, s/he may have decided to disregard this and suffice with the direct translation that still provides a digestible idea about what is taking place in the scene. Hence, the semantic equivalence errors here are minor.

**Table 6:** Quality assessment of the subtitles translated by direct translation.

Arabic	English Subtitle	Functional Equivalence	Acceptability	Readability
يا ريتني كنت معاهم	I wish I were with them	0.5 (semantic)	0	0
كنت عملت عمايل	I'd have done many things	0.5 (semantic)	0	0
يا ترى انت فين يا مرزوق؟	Where are you, Marzouq?	0.5 (semantic)	0	0

### 5.4 Generalization

When using generalization as a translation strategy, the translator replaces an ST cultural term with a less specific TT term to make the subtitle more comprehensible to the target audience. It can be done by using a superordinate term, or by paraphrasing, which is the most used generalization technique in the data analyzed.

Generalization was used to render terms with religious/cultural meanings/references. Table 7 shows how the expressions involving the words “ربنا” (God) and “الله” (Allah) were translated by less specific cultural references that omit the religious/cultural references and hence could read more natural to the target audience in this context. The same strategy was employed in translating the Islamic greeting commonly used in the ST culture “سلام عليكم” (Peace be upon you) and “سلام ورحمة الله وبركاته” (Allah's peace, mercy, and blessing). The religious reference in the greeting was replaced with a general greeting (Hello) that serves the same function and sounds more intelligible to the TT reader. This replacement with a more general term/a paraphrase is in uniform with previous subtitling attempts of religious terms to different languages [[28], [29], [30]].

**Table 7:** Generalization of religious terms.

Time	Arabic	English Subtitle	Back Translation
12:48	ربنا يخليك	Thanks a lot	May God let you live long.
12:54	ربنا يبارك فيكم	Thank you	May God bless you.
12:54	ربنا يخليك يا باشا	You're welcome, sir	May God let you live long, Pasha.
13:07	ربنا يخليك يا باشا	Thank you so much, sir	May God let you live long, Pasha.
15:00	ربنا ما يحرمنا منك	May you live a long and happy life	A thousand thanks, Pasha, may God don't deprive us of you.
27:06	سلام عليكم يا عم الحاج سلام ورحمة الله وبركاته	Hello, uncle! Hello	Peace be upon you, Uncle Hajj. Allah's peace, mercy, and blessing.
51:58	ربنا يديك الصحة	I wish you good health	May God give you health.
51:59	الحمد لله ربنا يديك طولة العمر	Thank God I wish you a long life	Thanks to Allah. May God give you a long life.
52:31	ان شاء الله هتسمعي اخبار حلوة دلوقتي	I hope you hear good news soon	If Allah wishes you will hear good news now.
52:51	ربنا يبارك فيك	Thanks	God bless you.
52:59	ماشي ربنا يبارك فيكم	Alright, wish you all the best	Alright, God bless you.
1:29:03	وإن شاء الله يكون خير يعني إن شاء الله	We hope he gets better soon	If Allah wishes he will be fine, if Allah wishes.

Generalization has also been employed to subtitle hyperbolic expressions that are culturally bound. Table 8 shows that the words “ده انت اللي منور” (It's you who lit us), “ألف شكر” (a thousand thanks), “ألف مبروك” (a thousand congratulations), “عشرة عمر” (lifetime companionship), and “١٠٠/١٠٠” (100/100) were replaced by more general terms that fit into the context in the TT. However, it is worth mentioning here that more accurate English equivalents are available for some hyperbolic expressions (e.g., a million thanks and lifelong friendship) which could have been used by the translator to render the same meaning into the English subtitle. However, there seems to be a hidden framework that the translators have been abiding by that entailed the use of a more formal subtitle rather than the less formal variety presented in the movie. This tendency towards formality is discussed in Section 5.7.

**Table 8:** Generalization of hyperbolic expressions.

Time	Arabic	English Subtitle	Back Translation
12:35	سعادة الباشا! منور يا باشا	Welcome sir, it's good to have you here	Pasha, your highness! You lit the place, Pasha!
12:48	نورتنا والله	It's a pleasure to have you here	You lit us, I swear to Allah.
12:50	ده انت اللي منور بزيارتك دي يا باشا	Thank you for coming sir	It's you who lit us with your visit, Pasha.
14:52	ألف شكر يا سعادة الريس، متشكرين	Thank you very much sir	A thousand thanks, Boss, your highness, thanks.
15:00	ألف شكر يا باشا	Thank you, sir.	A thousand thanks, Pasha, may God don't deprive us of you.
52:27	عشرة عمر	They are all old friends of mine	Lifelong companionship.
52:47	أخبارك ايه تمام؟ تمام ١٠٠/١٠٠؟	How you doing? How are you?	How are you? Good? 100/100?
1:33:49	حمدا لله على سلامته وألف مبروك	And congratulations on his safe return	Thanks to Allah for his safety and a thousand congratulations.

Table 9 summarizes the quality assessment of the subtitles where generalization is used. Many instances have a minor change in the style of the language used, where religious terms (e.g., “ربنا” (God)) and hyperbolic expressions (e.g., “ألف شكر” (a thousand thanks)) are rendered into more general and formal terms that are more acceptable for the TT reader despite losing some of their force. As noted above, this difference in style from informality in the ST to formality in the TT appears to be a strategy the translator follows in many instances in the data. Moreover, there are two instances of minor grammar errors, “How you doing?” misses the verb to be “are” and “Alright, wish you all the best” misses the subject “I”. Nonetheless, both instances are minor since, despite violating the conventions of standard written language, they are acceptable in spoken language and their meaning can be easily understood.

**Table 9:** Quality assessment of the subtitles translated by generalization.

Arabic	English Subtitle	Functional Equivalence	Acceptability	Readability
سعادة الباشا! منور يا باشا	Welcome sir, it's good to have you here	0.25 (stylistic)	0	0
ربنا يخليك	Thanks a lot	0.25 (stylistic)	0	0
نورتنا والله	It's a pleasure to have you here	0.25 (stylistic)	0	0
ده انت اللي منور بزيارتك دي يا باشا	Thank you for coming sir	0.25 (stylistic)	0	0
ربنا يبارك فيكم	Thank you	0.25 (stylistic)	0	0
ربنا يخليك يا باشا	You're welcome, sir	0.25 (stylistic)	0	0
ربنا يخليك يا باشا	Thank you so much, sir	0.25 (stylistic)	0	0
ألف شكر يا سعادة الريس، متشكرين	Thank you very much sir	0.25 (stylistic)	0	0
ألف شكر يا باشا	Thank you, sir.	0.25 (stylistic)	0	0
ربنا ما يحرمنا منك	May you live a long and happy life	0	0	0
سلام عليكم يا عم الحاج سلام ورحمة الله وبركاته	Hello, uncle! Hello	0	0	0
ربنا يديك الصحة	I wish you good health	0	0	0
الحمد لله ربنا يديك طولة العمر	Thank God I wish you a long life	0	0	0
عشرة عمر	They are all old friends of mine	0	0	0
ان شاء الله هتسمعي اخبار حلوة دلوقتي	I hope you hear good news soon	0	0	0
أخبارك ايه تمام؟ تمام ١٠٠/١٠٠؟	How you doing? How are you?	0.25 (stylistic)	0.25 (grammar)	0
ربنا يبارك فيك	Thanks	0	0	0
ماشي ربنا يبارك فيكم	Alright, wish you all the best	0	0.25 (grammar)	0
وإن شاء الله يكون خير يعني إن شاء الله	We hope he gets better soon	0.25 (stylistic)	0	0
حمدا لله على سلامته وألف مبروك	And congratulations on his safe return	0.25 (stylistic)	0	0

### 5.5 Substitution

A translator may choose to replace an ST cultural term with another term that is related to the TT culture or that is cross-cultural. This strategy usually requires much effort from the translator since it is more about bridging the gap between two cultures than translating word for word [17]. Despite that, substitution is found to be the most used translation strategy (27 occurrences) in the analyzed subtitles. This may prove that the translator was more oriented toward the TT.

Cultural substitution was mainly used when translating positive address tags that reflect respect for superiors or the elderly (Table 10). Terms like “سعادة الباشا” (Pasha, your highness), “عم الحج” (Uncle Hajj), “ريس” (Boss), and “حضرتك” (Your Presence) may not be easy to understand by the target audience. The translator, therefore, had to act by either attempting to substitute these tags with TT references, in this case, “sir” and “uncle”, or completely omitting these references (see Section 5.6). Although these substitutions do not reflect the sense of the ST references verbatim, they are considered more acceptable for TT readers. The same applies to the term of endearment “يا حبيبي يا صغير” (my little love) usually used with little ones in the ST culture, being translated as “dear”. Note also that the selected translations are found to have a more formal tone, which is also noticed in other instances in the TT as depicted in Section 5.7.

**Table 10:** Cultural substitution of positive address tags.

Time	Arabic	English Subtitle	Back Translation
12:35	سعادة الباشا! منور يا باشا	Welcome sir, it's good to have you here	Pasha, your highness! You lit the place, Pasha!
12:42	تعال يا عم الحج وسع	Please, make room, uncle	Come, Uncle Hajj, make room.
12:43	اتفضل يا باشا	Come on, sir	Come on, Pasha.
12:50	ده انت اللي منور بزيارتك دي يا باشا	Thank you for coming sir	It's you who lit us with your visit, Pasha.
12:54	ربنا يخليك يا باشا	You're welcome, sir	May God let you live long, Pasha.
13:51	بص يا سعادة الريس	Look, sir	Look, Boss, your highness.
14:52	ألف شكر يا سعادة الريس، متشكرين	Thank you very much sir	A thousand thanks, Boss, your highness, thanks.
15:00	ألف شكر يا باشا، ربنا ما يحرمنا منك	Thank you, sir. May you live a long and happy life	A thousand thanks, Pasha, may God don't deprive us of you.
27:06	سلام عليكم يا عم الحاج سلام ورحمة الله وبركاته	Hello, uncle! Hello	Peace be upon you, Uncle Hajj. Allah's peace, mercy, and blessing.
44:42	4 برجر وحاجة ساقعة. حاضر يا ريس	We'd like four hamburgers and cold drinks. Yes, sir	4 burgers and something cold. Yes, Boss.
1:15:33	مساء الخير يا ريس	Good evening, sir	Good evening, Boss.
1:22:10	عايزة من حضرتك خدمة	I want something from you, sir	I want a favor from Your Presence.
1:35:03	تعالى يا حبيبي يا صغير تعالى.	Come here, dear	Come my little love come.

Cultural substitution was also used when translating negative address tags for offending or reprimanding others (Table 11). Offenses like “رمة” (cadaver) and “يا ولاد البلغة” (sons of shoes) are conventional in the ST culture but unconventional in the TT culture. Therefore, they were both translated as “loser(s)”. Again, this translation might not exactly show the differences in the meaning of both terms of offense, but the translator may have considered this a negligible issue, focusing only on bringing to the TT reader's attention that there is an offense being uttered no matter what the offense is. The translator follows the same technique when translating the terms of reprimand “قرفتني معاك” (You disgusted me) and “غلبتني معاك” (You made me helpless), rendering both as “I'm sick of you”, a more acceptable expression for the target audience.

**Table 11:** Cultural substitution of negative address tags.

Time	Arabic	English Subtitle	Back Translation
14:49	خليك شاطر، متبقاش رمة زي أبوك	Be a good man. Don't be a loser like your father	Be clever, don't be a cadaver like your father.
18:17	قرفتني معاك، تعبتني معاك	I'm sick of you, man!	You disgusted me. You made me tired.
19:22	غلبتني معاك	I'm sick of you	You made me helpless.
1:22:27	يا ولاد البلغة	Losers!	Sons of shoes!

One final incident of cultural substitution in the analyzed subtitles is when the translator substituted the ST culturally bound birthday song with one that fits the TT culture (Table 12). In the ST culture, the common birthday song says “سنة حلوة يا جميل” (Have a good year you beautiful), but its equivalent in the TT culture says “Happy birthday to you”, which is considered a successful domestication of the popular song.

**Table 12:** Cultural substitution of the birthday song.

Time	Arabic	English Subtitle	Back Translation
14:07	سنة حلوة يا جميل، سنة حلوة يا جميل، يلا حالا بالا بالا حيوا أبو الفصاد	Happy birthday to you. Let's celebrate “Abu al-fasad's” birthday now	Have a good year you beautiful, have a good year you beautiful, let's now cheer for Abu al-fasad.

Another technique of the substitution strategy is substituting the ST culturally bound reference into a paraphrase that has a different sense but fits the situation at hand. Pedersen states that this situational paraphrase acts as “a quasi-omission strategy” [17, p. 95] since the ST term is omitted but not completely since it is replaced with a term/expression that carries a different meaning but makes the situation more comprehensible to the TT reader.

In the analyzed data, substitution by situational paraphrase was used when translating some expressions of greeting (Table 13). For instance, in the ST culture, it is common to reply to the greeting “ازيك عامل ايه؟” (How are you?) by “كتر خيرك” (May you do more goodness). This would not sound natural to the TT reader's ear. Therefore, the translator substituted the ST term with a more common reply (I'm fine, thanks). This translation does not render the sense in the ST term, but it fits the situation well. Another example is the use of “كل سنة وحضرتك طيب” (May you stay kind every year) and its reply “وانت بالصحة والسلامة” (May you have health and safety). These greetings are usually used in any celebration or feast in the ST culture. However, the TT reader may find them confusing, so the translator resorted to other expressions that align with the situation, which are “Thanks for coming” and “I'm happy to be here”.

**Table 13:** Substitution by situational paraphrase of greetings.

Time	Arabic	English Subtitle	Back Translation
12:37	ازيك عامل ايه؟ عامل ايه يا باشا كتر خيرك يا باشا	How are you? I'm fine, thanks	How do you do? How do you do, Pasha. May you do more goodness, Pasha.
12:56	كل سنة وحضرتك طيب	Thanks for coming	May you stay kind every year.
12:56	وانت بالصحة والسلامة	I'm happy to be here	May you have health and safety.

Moreover, situational paraphrase took place when translating some negative address tags, as seen in Table 14. The terms “انت مخاوي” and “انت ملبوس” mean that someone is possessed by a paranormal being or power. These terms are ordinary in the ST culture, but they may not be so in the TT culture. Therefore, the translator substituted them with “charlatan” and “fraud”, which both have a different sense (being an imposter or false) but can go with their context.

**Table 14:** Substitution by situational paraphrase of negative address tags.

Time	Arabic	English Subtitle	Back Translation
18:52	انت مخاوي، انت نصاب	You're a charlatan, you're a fraud	You're possessed. You're an imposter.
19:01	انت ملبوس	You're a charlatan	You're possessed.
19:01	انت ملبوس	You're a fraud	You're possessed.

Another use of situational paraphrase was found in the translation of some hyperbolic expressions (Table 15). The translation of “والله يعني زارنا النبي” (I swear to Allah, the Messenger visited us) and “عقبال 100 سنة” (May he live 100 years) misses the hyperbolic force in the ST terms where more neutral terms (“Thank you very much” and “Happy birthday”, resp.) are used. The translator, however, might have decided to do so since it is hardly unlikely that the target audience will not notice any discrepancy between the translations and the scenes they portray, not to mention the word economy achieved for the TT subtitles.

**Table 15:** Substitution by situational paraphrase of hyperbolic expressions.

Time	Arabic	English Subtitle	Back Translation
12:50	والله يعني زارنا النبي	Thank you very much	I swear to Allah, the Messenger visited us.
13:07	يا عم عقبال 100 سنة	I wish him a happy birthday	May he live 100 years, uncle.
14:47	عقبال 100 سنة	Happy birthday to you	May you live 100 years.

Table 16 summarizes the quality assessment of the subtitles where substitution is used. There are a number of functional equivalence errors spotted in the subtitles translated by substitution. One recurring error is the change in the style of many address tags from informal, culturally specific references in the ST to more TT-oriented tags, with most having a more formal tone (e.g., substituting “باشا” with “sir”). This error is minor, however, since the general meaning of the utterances is retained. On the other hand, the translation of a few negative address tags shows a semantic equivalence error. This difference in the meaning of the ST terms “رمة”, “مخاوي”, and “ملبوس” and their translations “loser”, “charlatan”, and “fraud” can be obvious to the ST reader. However, to the TT reader, the English words go well with each situation. Although a more accurate translation could have been provided (i.e., using “possessed” which is closer in meaning to the ST terms instead of “fraud” and “charlatan”), these errors remain minor in effect. The same applies to the translation of some expressions of greeting in the ST (e.g., “والله يعني زارنا النبي” (I swear to Allah, the Messenger visited us) and “كل سنة وحضرتك طيب” (May you stay kind every year)). The translator decided to sacrifice these culturally specific references and replace them with other references that sound more natural to the target audience (e.g., “Thank you very much” and “Thanks for coming”), where there is a change in meaning that does not affect the general idea or flow of speech.

**Table 16:** Quality assessment of the subtitles translated by substitution.

Arabic	English Subtitle	Functional Equivalence	Acceptability	Readability
سعادة الباشا! منور يا باشا	Welcome sir, it's good to have you here	0.25 (stylistic)	0	0
ازيك عامل ايه؟ عامل ايه يا باشا كتر خيرك يا باشا	How are you? I'm fine, thanks	0	0	0
تعال يا عم الحج وسع	Please, make room, uncle	0.25 (stylistic)	0	0
اتفضل يا باشا	Come on, sir	0.25 (stylistic)	0	0
ده انت اللي منور بزيارتك دي يا باشا	Thank you for coming sir	0.25 (stylistic)	0	0
والله يعني زارنا النبي	Thank you very much	0.5 (semantic)	0	0
ربنا يخليك يا باشا	You're welcome, sir	0.25 (stylistic)	0	0
كل سنة وحضرتك طيب	Thanks for coming	0.5 (semantic)	0	0
وانت بالصحة والسلامة	I'm happy to be here	0.5 (semantic)	0	0
يا عم عقبال 100 سنة	I wish him a happy birthday	0.5 (semantic)	0	0
سنة حلوة يا جميل، سنة حلوة يا جميل، يلا حالا بالا بالا حيوا أبو الفصاد	Happy birthday to you. Let's celebrate “Abu al-fasad's” birthday now	0	0	0
بص يا سعادة الريس	Look, sir	0.25 (stylistic)	0	0
ألف شكر يا سعادة الريس، متشكرين	Thank you very much sir	0.25 (stylistic)	0	0
عقبال 100 سنة	Happy birthday to you	0.5 (semantic)	0	0
خليك شاطر، متبقاش رمة زي أبوك	Be a good man. Don't be a loser like your father	0.5 (semantic)	0	0
ألف شكر يا باشا، ربنا ما يحرمنا منك	Thank you, sir. May you live a long and happy life	0.25 (stylistic)	0	0
قرفتني معاك، تعبتني معاك	I'm sick of you, man!	0	0	0
انت مخاوي، انت نصاب	You're a charlatan, you're a fraud	0.5 (semantic)	0	0
انت ملبوس	You're a charlatan	0.5 (semantic)	0	0
انت ملبوس	You're a fraud	0.5 (semantic)	0	0
غلبتني معاك	I'm sick of you	0	0	0
سلام عليكم يا عم الحاج سلام ورحمة الله وبركاته	Hello, uncle! Hello	0.25 (stylistic)	0	0
4 برجر وحاجة ساقعة. حاضر يا ريس	We'd like four hamburgers and cold drinks. Yes, sir	0.25 (stylistic)	0	0
مساء الخير يا ريس	Good evening, sir	0.25 (stylistic)	0	0
عايزة من حضرتك خدمة	I want something from you, sir	0	0	0
يا ولاد البلغة	Losers!	0.25 (stylistic)	0	0
تعالى يا حبيبي يا صغير تعالى.	Come here, dear	0	0	0

### 5.6 Omission

Omission is a valid strategy used by translators. It involves removing the ST cultural reference without reproducing it in any way in the TT [25]. The terms omitted in the present study belong to three categories: first, positive address tags that speakers use in slang to address each other, rather than using names or family names, to “pertain the feeling of importance, intimacy and self-confidence” [31, p. 80]; second, religious terms, whose translation has been always problematic [32]; and third, hyperbolic expressions unique to a culture in terms of formulae or sense [33]. Consider the examples in Table 17 extracted from the movie.

**Table 17:** Omission of positive address tags.

Time	Arabic	English Subtitle	Back Translation
12:37	ازيك عامل ايه؟ عامل ايه يا**باشا** كتر خيرك يا**باشا**	How are you? I'm fine, thanks	How do you do? How do you do, Pasha. May you do more goodness, Pasha.
12:40	يا**باشا** اتفضل اتفضل	Come inside, please	Come in, Pasha, come in.
12:45	اتفضل اتفضل يا**باشا**	Come on in	Come on, come on, Pasha.
13:07	يا**عم** عقبال 100 سنة	I wish him a happy birthday	May he live 100 years, uncle.
19:01	متتكلميش بالطريقة دي يا **أمي** لو سمحتي	Don't talk to me this way	Don't talk to me this way, my mother, please.
19:09	معلش يا **أمي** أنا آسف	I'm sorry	Excuse me, my mother, I'm sorry.
19:14	مفيش حاجة يا**عم**	Nothing	Nothing, uncle.
21:02	مش طالع يا **عم**	I won't go anywhere!	I'm not going out, uncle!
44:36	خد **ياض**	Hey	Come, boy.
52:06	هات كوكاكولا تانية هنا **يابني**	Give me another Coca-Cola	Bring another Coca-Cola here, my son.
52:12	اتفضل يا **عمي**	Here you go	Here you go, my uncle.
52:19	اشربي يا **بنتي**، كلي	Drink and have some of this	Drink, my daughter, eat.
1:13:00	الواد هيشتغل هنا ممنوع يا **ستي**ممنوع	This boy is going to work here. Women are not allowed	The boy is going to work here. It's forbidden, my lady, forbidden.
1:15:42	أتمنى مكونش أزعجت **سيادتك**	I hope I didn't bother you	I hope I didn't bother your highness.
1:15:54	عايزين بس خدمة بس من **حضرتك**	We're here to ask you for a favor, please	We just want a favor from Your Presence.

As seen in Table 17, the translator removed terms of address, such as “باشا” (Pasha), “أمي” (mother), “بنتي” (daughter), and “سيادتك” (your highness), and depended on the context to convey the meaning. The decision of the translator could be justified by many reasons; it could be because s/he was trying to avoid redundancy in reusing some terms (e.g., “sir” which was used in many incidents to render “باشا” (Pasha) into English; see Section 5.5), or it could be because the translator was trying to stop a problematic foreign term from entering the TT in form (e.g., “بنتي” (daughter)) to refer to a young woman or in a context where it does not fit because of its in/formality (e.g., “سيادتك” (your highness)). These omissions, however, did not seem to affect the meaning intended.

In Table 18, the religious term “والله” (I swear to Allah) was omitted. Perhaps the choice was made by the translator because of its cultural, yet peripheral nature in the ST. That is, the term “والله” is not central in the ST and its removal does not affect the meaning in either the ST or the TT (despite affecting the force of the utterance). By removing it, the translator probably saved him/herself from having to include a term with a religious reference that may be, generally or contextually, unknown to the target audience.

**Table 18:** Omission of religious terms.

Time	Arabic	English Subtitle	Back Translation
12:48	نورتنا **والله**	It's a pleasure to have you here	You lit us, I swear to Allah.
12:50	**والله** يعني زارنا النبي	Thank you very much	I swear to Allah, the Messenger visited us.
51:56	واحشني **والله**	I miss you, how are you?	I miss you; I swear to Allah.
52:50	انت وحشني **واللهوحشني والله**	I wanted to see you	I miss you; I swear to Allah. I miss you; I swear to Allah.
1:33:49	حمدا لله على سلامته وألف مبروك	And congratulations on his safe return	Thanks to Allah for his safety and a thousand congratulations.

Table 19 below shows how the translator may opt for translating ST culturally specific hyperbolic expressions by means of omission. The term “أ**لف مرة**” (a thousand times) does not have a literal counterpart in the TT in this context [33]. Its retention may have resulted in a strange subtitle, and providing its equivalence in the TT could have led to a very long subtitle given the length of the utterance at hand.

**Table 19:** Omission of hyperbolic expressions.

Time	Arabic	English Subtitle	Back Translation
1:09:37	ما انتي عارفة يا ست القانون عندنا ميسمحش بتشغيل سيدات وقلتلك الكلام ده قبل كده أ**لف مرة**	I have already told you that women aren't allowed to work here	You know, lady, law here does not allow women labor, and I told you this before a thousand times.

Table 20 summarizes the quality assessment of the subtitles where omission is used. The main error spotted is stylistic, where the omission of ST terms has led to a change in the style and tone of the rendered TT. For example, the omission of the informal address tags “باشا” and “عم” has made the rendered text more formal. The same applies to the omission of the religious term “الله” and the hyperbolic expression “ألف مرة”, which makes the rendered TT more neutral and less culturally specific. Nevertheless, these errors are minor since there is no hindrance in understanding on the part of the target audience.

**Table 20:** Quality assessment of the subtitles translated by omission.

Arabic	English Subtitle	Functional Equivalence	Acceptability	Readability
ازيك عامل ايه؟ عامل ايه يا**باشا** كتر خيرك يا**باشا**	How are you? I'm fine, thanks	0.25 (stylistic)	0	0
يا**باشا** اتفضل اتفضل	Come inside, please	0.25 (stylistic)	0	0
اتفضل اتفضل يا**باشا**	Come on in	0.25 (stylistic)	0	0
نورتنا **والله**	It's a pleasure to have you here	0.25 (stylistic)	0	0
**والله** يعني زارنا النبي	Thank you very much	0.25 (stylistic)	0	0
يا**عم** عقبال 100 سنة	I wish him a happy birthday	0.25 (stylistic)	0	0
متتكلميش بالطريقة دي يا **أمي** لو سمحتي	Don't talk to me this way	0.25 (stylistic)	0	0
معلش يا **أمي** أنا آسف	I'm sorry	0.25 (stylistic)	0	0
مفيش حاجة يا**عم**	Nothing	0.25 (stylistic)	0	0
مش طالع يا **عم**	I won't go anywhere!	0.25 (stylistic)	0	0
خد **ياض**	Hey	0.25 (stylistic)	0	0
واحشني **والله**	I miss you, how are you?	0.25 (stylistic)	0	0
هات كوكاكولا تانية هنا **يابني**	Give me another Coca-Cola	0.25 (stylistic)	0	0
اتفضل يا **عمي**	Here you go	0.25 (stylistic)	0	0
اشربي يا **بنتي**، كلي	Drink and have some of this	0.25 (stylistic)	0	0
انت وحشني **واللهوحشني والله**	I wanted to see you	0.25 (stylistic)	0	0
ما انتي عارفة يا ست القانون عندنا ميسمحش بتشغيل سيدات وقلتلك الكلام ده قبل كده أ**لف مرة**.	I have already told you that women aren't allowed to work here	0.25 (stylistic)	0	0
الواد هيشتغل هنا ممنوع يا **ستي**ممنوع	This boy is going to work here. Women are not allowed	0	0	0
أتمنى مكونش أزعجت **سيادتك**	I hope I didn't bother you	0.25 (stylistic)	0	0
عايزين بس خدمة بس من **حضرتك**	We're here to ask you for a favor, please	0.25 (stylistic)	0	0
حمدا لله على سلامته وألف مبروك	And congratulations on his safe return	0.25 (stylistic)	0	0

**Fig. 2:**
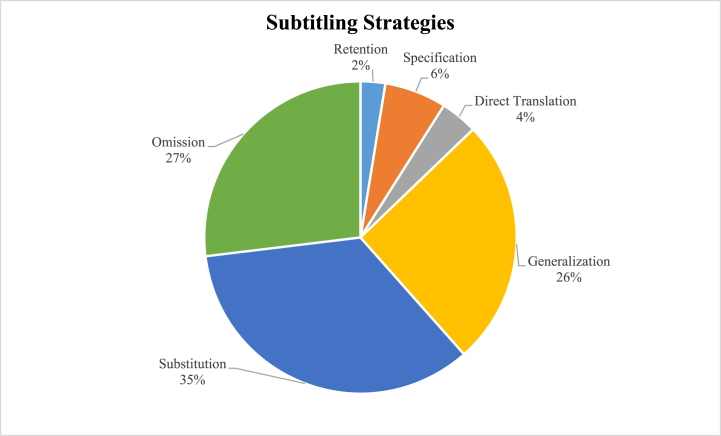
The subtitling strategies used in the data.

To conclude, Fig. 2 provides an overall view of the prevalence of using each subtitling strategy in the data. As seen in the pie chart, the three most used strategies are substitution, omission, and generalization, respectively.

### 5.7 Formality

Pedersen [17,25] tackled formality from the angle of being a media-specific-constraint influencing parameter rather than a translation strategy in subtitling culturally specific references. He confirmed that since subtitles are written, they tend to be more formal than the spoken utterance. He added that translators may opt to render cultural references formally because of the lack of transculturality between the ST and the TT and/or a limited space to provide additions/paraphrases. However, the data analyzed here suggest otherwise; the subtitles seem to show a continuous strategic use of formality, as a secondary strategy accompanying other strategies, to render the Arabic speech to English subtitles, despite the intercultural nature of many cultural references and the resulting lengthy translations.

To illustrate, the hyperbolic ST term “ألف شكر” (a thousand thanks) was translated formally as “Thank you very much” despite having a shorter TT equivalent idiom “Thanks a million” which was probably not chosen for its informality. The same strategic tendency to choose a formal variety was evident in “برجر وحاجة ساقعة 4” (4 burgers and something cold), translated as “We'd like four hamburgers and cold drinks”, where the translator made the subtitle much longer by adding a formal phrase for request “We'd like” instead of the informal “Four hamburgers and cold drinks” which could have been understood by the target audience and follows subtitles' space constraints. Formality was also evident structurally in translating “نورتنا والله” (You lit us, I swear to Allah) into “It's a pleasure to have you here” despite the availability of the shorter less formal version in the TL “Pleasure to have you”. Formality as a strategy was also evident in avoiding translating some informal/common beliefs or cultural themes. The translator subtitled “انت مخاوى” and “انت ملبوس”, which mean that someone is possessed by an evil spirit, by the terms, “fraud” and “charlatan”, which are more formal and lack the negative connotative references of the ST utterance.

This use of formality is, hence, a strategy that can be used by translators to render cultural references. This partially agrees with [12] that suggested formality as a translation strategy, but this paper adds that formality must be accompanied by other strategies.

## 6 Conclusion

This study aimed to identify and assess the subtitling strategies used in translating cultural references in the award-winning Egyptian movie *Feathers* released in 2021. To this end, Pedersen's (2011) framework for subtitling cultural references along with Pedersen's (2017) quality assessment FAR model was used to analyze the data. The results revealed that all Pedersen's suggested strategies, except for using an official equivalent, were used, but with frequencies/occurrences different from his. That is, substitution was the most frequently used strategy rather than retention as argued by Pedersen. Moreover, an additional complementary strategy was identified: using formal language to render informal language. This strategy was used despite the availability of a shorter register-specific option in the TT. The choice could probably be because the translator tried to present language that is more likely to occur in these contexts, thus sacrificing brevity and register for naturalness. The quality assessment showed that most subtitles were of good quality with few minor errors mostly related to formally rendering informal subtitles.

This study encountered certain limitations that warrant acknowledgment. Firstly, the assessment of the collected data inherently involved a high degree of subjectivity, as is characteristic of qualitative research methodologies. However, to mitigate this potential bias, inter-rater reliability measures were implemented to ensure consensus and consistency in the evaluation of the data. Furthermore, it should be noted that the chosen film presented multiple versions featuring distinct sets of subtitles. To ensure precision and reliability, the version encompassing professionally executed subtitles, as provided by an established agency, was selected. This approach aimed to avoid potential inaccuracies that could arise from the efforts of amateur translators or fansubbing.

The primary objective of this study was to serve the interests of professional translators by exploring and evaluating the diverse strategies employed in the translation of culturally specific references from a culturally bound dialect (Egyptian Arabic) into English. The findings and insights derived from this research endeavor have the potential to guide practitioners in shaping their own methodologies, facilitating the transmission of an authentic essence from the source text to the target audience. Future studies conducted in the English language should be undertaken to investigate further the strategies employed and the overall quality of subtitling from Arabic into English. It is also recommended to have multimodal translation studies that shed light on the role of metalinguistic features influencing the subtitling process.

## Author contribution statement

All authors listed have significantly contributed to the investigation, development and writing of this article.

## Data availability statement

Data included in article/supplementary material/referenced in article.

## Additional information

No additional information is available for this paper.

## Declaration of competing interest

The authors declare that they have no known competing financial interests or personal relationships that could have appeared to influence the work reported in this paper.
